# Topics of Public Interest

**Published:** 1906-10

**Authors:** 


### TOPICS OF PUBLIC INTEREST.

### Interstate Reciprocity in Medical Licensure

THE exchange of licenses to practise medicine between the states has attracted public interest again, because some of those uninformed by previous discussion of the subject in medical journals and medical licensing boards have dealt with it *de novo*,—as though nothing had ever been said on the question, or as if only sophomores had spoken upon it. New York, in particular, has been criticised because it would not lower its standards, violate its medical laws, and throw open its doors to all who would enter.

These critics would have the Empire State exact less of those from other political divisions who would come within its borders to practise medicine, than from its own citizens, an inconsistency,—yes, even an absurdity,—that is apparent on its face. The principles of reciprocity have been settled long ago and so clearly that he who runs may read. They are, in brief, equality of preliminary requirements, uniformity of collegiate training, and similarity in state examinations. Whenever the medical colleges awaken to the fact that they must agree upon a common minimum standard of entrance requirements of the medical student, as well as a uniformity of methods in training, including length of time required to obtain the doctorate degree, then the examining boards, perforce, will adopt equality of standards and reciprocity will become an established fact. It has been claimed by some who do not quite understand the situation that the state examining boards are responsible in the matter of reciprocity, whereas the medical colleges are the real and most important factor in the case. The boards do not make but merely execute the laws of the states. The medical colleges make their own rules and are responsible for the lack of uniform standards. Is it to be expected that the boards will create a uniformity in finals before the colleges agree upon equality of preliminaries and academic methods?

As a contribution to the general subject, bringing out many facts that have developed in the course of the negotiations between New York and other states looking to the establishment of reciprocity, or in the course of the administration of agreements that have already become operative, the subjoined letter of Dr. Rogers becomes of interest:

STATE OF NEW YORK
EDUCATION DEPARTMENT
ALBANY.

SEPTEMBER 18, 1906.

J. B. GREGG CUSTIS, M.D., PRESIDENT
BOARD OF SUPERVISORS IN MEDICINE AND PHARMACY
WASHINGTON, D. C.

DEAR SIR:

I beg to acknowledge the receipt of your communication of August 29 in regard to reciprocity in medical licensure between New York and the District of Columbia. The facts cited in your letter are matters of record in this Department and I hasten to assure you of our sincere hope that in the near future reciprocity between New York and the District of Columbia may become an established fact. Out of our experience in attaining reciprocity between New York and the states of New Jersey, Michigan and Ohio, we are convinced that a conference of representatives of the

District and State is essential to the perfecting of the details necessary for such reciprocal relations. We will gladly appoint representatives to confer with your representatives in the near future at a time and place convenient to both.

Meanwhile, I invite your consideration of the following facts which have developed in our administration of the reciprocal relations recently entered into between New Jersey, Michigan and Ohio. The details of reciprocity in medical licensure are not clearly understood.

*First illustration, New York and Ohio.* The clause of the New York statute which permits of reciprocity reads as follows:

"Applicants examined and licensed by other state examining boards registered by the Regents as maintaining standards not lower than those provided by this article . . . may without further examination, on payment of $10 to the Regents and on submitting such evidence as they may require, receive from them an indorsement of their licenses or diplomas conferring all rights and privileges of a Regents license issued after examination.

The amendment of the Ohio statute which rendered reciprocity with New York possible, reads:

"The Board may in its discretion dispense with an examination in the case of a physician or surgeon duly authorized to practise medicine or surgery in any other state . . . who may desire to change his residence to and practice his profession in Ohio, and who makes application on a form to be prescribed by the Board . . . and presents a certificate or license issued by the medical board of such state . . . accorded only to applicants from states . . . whose laws demand qualifications of equal grade with those required in Ohio, but such examination shall not be dispensed with unless under the law and regulations of the state . . . equal rights and privileges are accorded to physicians and surgeons of Ohio holding the certificate of the Board who may desire to remove to and practice in such state . . ."

It will be noted by reference to the agreement with Ohio that the first article states that the basis on which reciprocity shall obtain between the states of Ohio and New York shall be a license earned on examination in either one of the states. After this agreement had been formally ratified by the signatures of the conferees and the agreement had gone into effect it transpired that the Ohio authorities under the clause "may dispense with" were about to license practitioners in Ohio that had never passed an examination because at the date of their registration in another state the Ohio statute required no examination. It was at once apparent that such action would negative the reciprocal relations, for such standards are lower than those provided by the New York statute which requires examination in all cases.

The basis of agreement provides that before the date it becomes effective the requirements of either state are to be determined by the requirements at the dates of licensure, but that subsequent to the date of agreement each state must maintain standards not lower than those provided by the statute of the other state.

*Second illustration, New York and Michigan.* While it appears above that each state must maintain standards not lower than the other, it does not preclude one state exacting higher; for example, the Michigan board wished to maintain a higher general preliminary requirement for admission to the medical schools and the licensing examination, and the applicants for admission to the Michigan licensing examination subsequent to Aug. 1, 1906, must not only present evidence of graduation from a four year high school course subsequent to an eight year preacademic course, but in such high school course he must have pursued certain prescribed studies such as English, mathematics, Latin and science, with the possibility of electing the remaining requirements from other specific subjects. Hence the Board of Supervisors in Medicine and Pharmacy of the District of Columbia can exact higher examination requirements if they deem wise, but whether an examination is lowered by the choice of questions allowed a candidate or improved, is a debatable subject and can very properly be referred to the conference for consideration.

*Third illustration, New York and New Jersey.* From the experience with New Jersey it is clearly apparent that Notes C and D of the "Law Regulating the Issue of License in the District of Columbia without Examination" can not continue in effect after reciprocity is entered into between New York and the District. For example, assume that reciprocity was entered into between New York and the District on August 1, 1906; that a candidate applying to the District Board is to be considered simply on his own merits; that he presents a copy of the license under which he has practised, shows evidence of moral character and of residence in the jurisdiction which granted his license for at least two years, all over the signature of the president and secretary of the board which granted his license; that he presented no evidence of a general preliminary education prior to his admission to the medical school from which he received his degree. The District Board licenses him on September 1 without examination. Two years later he presents that license to New York State for indorsement, which can not be done under the New York statute. *It was not "issued after examination."*

Suppose another case, however, in which the District Board examined the candidate on Sept. 1, 1906, but did not require evidence of the general preliminary education required by the New York statute. For example, in the fall of 1899 a New York student presents evidence that he had completed two years of high school work; was given credit for the same and told that he must work off two years more of high school work for admission to his first medical year, provided that the medical school might condition him in subjects aggregating not more than one year of high school preparation. That instead of entering the New York medical school in 1901 and graduating therefrom in 1905, he avoids the New York requirement by going to a Maryland medical school, secures his degree in 1903, presents his credentials to the District Board, passes their examination in 1906, and is licensed in October. That two years later he presents said license to New York for endorsement. The New York Board is debarred from licensing him by a provision of the statute which says that "The New York medical schools and the New York medical students shall not be discriminated against by the registration of any medical school out of the state whose minimum graduation standard is less than that fixed by statute for New York medical schools.

He at once enters the courts to compel the indorsement of his license by the New York Board. The court is confronted with this question in logic. (1) The District Board grants to the New York licentiate privileges equivalent to those the applicant seeks. (Note D, District Law). (2) The Regents have registered the District as maintaining standards not lower than those provided by the New York statute. (Agreement). (3) Therefore the New York Board must indorse the applicant's license. (Decision).

This is not a theoretical or an academic question. While New York will gladly enter into reciprocal relations with all states of the Union that will maintain standards not lower than those provided by the New York statute, she should not enter into relations that will involve expensive legal proceedings to weaken the standards that have been attained through many years by wise statute and careful administration—standards that are recognised by the medical profession, medical schools and the educational sentiment of the State as eminently practicable, helpful and uplifting.

Yours very respectfully

(Signed) HOWARD J. ROGERS,

First Assistant Commissioner of Education.

